# Recent Advances of Interface Exciplex in Organic Light-Emitting Diodes

**DOI:** 10.3390/mi13020298

**Published:** 2022-02-14

**Authors:** Jianhua Shao, Cong Chen, Wencheng Zhao, Erdong Zhang, Wenjie Ma, Yuanping Sun, Ping Chen, Ren Sheng

**Affiliations:** 1 Institute of Physics and Electronic Information, Yantai University, Yantai 264005, China; jianhuashaotyu@outlook.com (J.S.); congchentyu@outlook.com (C.C.); wenchengzhao@outlook.com (W.Z.); erdong25ytu@hotmail.com (E.Z.); mwjytu@gmail.com (W.M.); ypsun@ytu.edu.cn (Y.S.); 2 State Key Laboratory on Integrated Optoelectronics, College of Electronic Science and Engineering, Jilin University, Changchun 130012, China

**Keywords:** organic light-emitting diodes, exciplex, phosphorescent, high efficiency, energy transfer

## Abstract

The interface exciplex system is a promising technology for reaching organic light-emitting diodes (OLEDs) with low turn-on voltages, high efficiencies and long lifetimes due to its unique virtue of barrier-free charge transport, well-confined recombination region, and thermally activated delayed fluorescence characteristics. In this review, we firstly illustrate the mechanism frameworks and superiorities of the interface exciplex system. We then summarize the primary applications of interface exciplex systems fabricated by doping and doping-free technologies. The operation mechanisms of these OLEDs are emphasized briefly. In addition, various novel strategies for further improving the performances of interface exciplex-based devices are demonstrated. We believe this review will give a promising perspective and attract researchers to further develop this technology in the future.

## 1. Introduction

As global climate warming and energy shortages become aggravated, developing lighting technology with energy conservation and environmental protection becomes a significant world issue [1]. Organic light-emitting diodes (OLEDs) have caught the attention of academia and industry due to their high performance, low power, and flexibility, which exhibit great potential for display and lighting applications [2,3,4,5,6]. In order to overcome the intrinsic low internal quantum efficiency (IQE) of fluorophores-based OLEDs, phosphorescent OLEDs are proposed as a promising technology due to their singlet and triplet harvesting properties [7]. These devices generate phosphorescence emission via the radiative transition process from T_1_ excited state to ground state S_0_, which can be enhanced by the intersystem crossing (ISC) process from S_1_ to T_1_ [8,9,10]. However, phosphorescent dyes are mostly organometallic complexes that contain heavy metal elements of platinum and iridium, increasing the manufacturing costs of commercialization [11].

In 2012, thermally activated delayed fluorescence (TADF) materials were first reported by the Adachi group, and are regarded as new generation emitters by academia and industry [12,13]. Such materials have a small singlet–triplet energy gap (Δ*E_ST_*), so that triplet excitons can be upconverted into singlet excitons via reverse intersystem crossing (RISC) at room temperature, thereby generating delayed fluorescence [14,15,16]. Unlike TADF material based on intramolecular charge transfer, the TADF process in exciplex should result from intermolecular charge transfer, which can noticeably improve the utilization of triplet excitons and enhance device efficiency [17,18]. Therefore, exciplexes can be employed as efficient TADF emitters or TADF hosts for dopants with satisfied charge transport properties. In general, exciplex architecture can be constructed by two strategies: (i) mixing the donor material and the acceptor material into the same layer to form bulk exciplex [19]; (ii) separating the donor layer and the acceptor layer to generate exciplex at the interface [20]. Meanwhile, interface exciplex-type OLEDs exhibit many advantages, such as simplified device architectures and lower driving voltages [21,22]. Nowadays, efficient OLEDs based on exciplex are widely reported, and EQEs of over 20% are realized in interface exciplex-based devices [23].

In this review, we emphatically describe the progress of interface exciplex-based OLEDs. The operating mechanism of interface exciplex is first demonstrated, then the design strategies and applications of interface exciplex fabricated by doping and doping-free technologies are summarized, including monochromatic and white OLEDs. Finally, an outlook for the improvement of interface exciplex-based OLED is proposed.

## 2. Basic Concept of Interface Exciplex

### 2.1. Interface Exciplex as Emitter

Exciplex emission is a product of electron transition from the lowest unoccupied molecular orbital (LUMO) of acceptor (A) to the highest occupied molecular orbital (HOMO) of the donor (D) [24,25]. The charge transfer (CT) excited state formed between the donor and acceptor layer under photoexcitation can be described as follows [26]:

D + A + *hν* → D* + A or D + A* → (D^δ+^A^δ−^)* → *hν*_ex_ + D + A (1)

**Figure 1:**
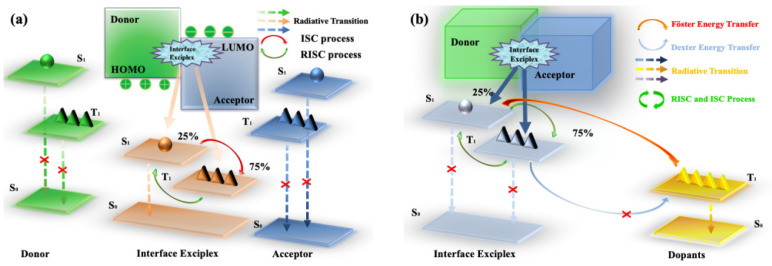
(**a**) Diagram of interface exciplex emission. (**b**) The energy transfer diagram of OLED based on interface exciplex host.

The emission peak of exciplex always exhibits a red shift compared with those of pure donor and acceptor [27,28,29,30,31,32,33,34,35,36,37], which can be expressed as follows [38]:

*hν*_max_ ≅ I_D_ − A_A_ − E_C_ (2)

where I_D_ is the ionization potential of the donor, A_A_ is the electron affinity of the acceptor, and E_C_ is the coulombic attraction energy. Figure 1a shows the interface exciplex emission process in the EL process. Spin statistics demonstrate that the proportion of 1:3 of singlet excitons to triplet excitons can be generated by electron-hole recombination [39]. In general, a significant energy-level bias between the HOMO of the donor and the LUMO of the acceptor is more likely to provide a small Δ*E_ST_* [40]. As for exciplex, the HOMO and LUMO are separated between donor and acceptor molecules, resulting in a small energy gap between singlet level and triplet level [40,41]. A small Δ*E_ST_* is essential for exciplex to obtain TADF properties, because minor Δ*E_ST_* allows triplet excitons to be upconverted into singlet excitons under thermal excitation via RISC, leading to the efficient radiative transition of singlet excitons [42]. The RISC rate (*k_RISC_*) in the interface exciplex system can be expressed by the Arrhenius equation [43]:

(3) $$k_{RISC}=Aexp\left(-\frac{\Delta E_{ST}}{kT}\right)$$

where *A* is frequency factor, *k* is the boltzmann constant, and *T* is the temperature. It should be noted that higher *k_RISC_* can be achieved when Δ*E_ST_* is smaller than *kT*, which is approximately 26 meV at 300 K [43]. One must note that, owing to large energy level bias between HOMO of the donor and LUMO of the acceptor, the main recombination zone of charge carriers can be confined at the heterojunction [44]. Moreover, for achieving an efficient interface exciplex emitter, the triplet energy level (T_1_) of exciplex should be lower than either the donor or the acceptor, so that the reverse energy transfer from exciplex to constituent molecular can be prevented [45,46,47,48].

### 2.2. Interface Exciplex as Host

It should be briefly pointed out that the energy-loss pathways in the interface exciplex system can be attributed to the following: (i) owing to the narrow exciton distribution area of the interface exciplex, the concentration quenching effect of triplet excitons is inevitable, especially at high current density [49]; (ii) the RISC rate is always lower than the ISC rate in the interface exciplex system, leading to the non-radiative transition processes of triplet excitons [50,51,52]. It is generally believed that using interface exciplex as an energy transfer host for dye is an effective way to improve the utilization of exciplex triplet excitons. Because the energy transfer process from exciplex to dopant is competitive with the energy-loss pathways [51], an efficient energy transfer process can effectively guard against the waste of interface exciplex triplet excitons. For serving as a host, the T_1_ of the exciplex should be higher than that of the dopants to prevent reverse energy transfer. Furthermore, the emission spectrum of exciplex should overlap well with the absorption spectrum of the emitter to achieve efficient energy transfer. The expression of energy transfer efficiency (Φ_ET_) is [52]:

(4) $$\Phi_{\mathrm{ET}}=\frac{k_{\text{ex-g}}}{k_{r}+k_{\mathrm{nr}}+k_{\text{ex-g}}}=\frac{k_{\text{ex-g}}}{(\frac{1}{\tau })+k_{\text{ex-g}}}$$

where k_ex-g_ is the energy transfer rate from the host of exciplex to the emitter, k_r_ is the radiative decay rate of exciplex, k_nr_ is the non-radiative decay rate of exciplex, and τ is the delay time of exciplex. As can been seen, higher Φ_ET_ can be reached with improved τ.

The energy transfer process in OLEDs based on interface exciplex is shown in Figure 1b. For doping-free OLEDs, the emission layer (EML) and the interface of exciplex are separated by the interlayer so that the short-range Dexter excitation transfer (DET) from exciplex to EML will be hindered. The unutilized triplet excitons can be upconverted into singlet excitons via an effective RISC process, and then the long-range Förster resonance energy transfer (FRET) can be enhanced, thereby improving the energy transfer to the EML [53,54]. As for doped OLEDs, the energy transfer from interface exciplex to dopant is greatly affected by the doping concentration [46,55]. Increasing doping concentration can improve the device efficiency in a reasonable concentration range [56]. However, with doping concentration increasing, the distance between interface exciplex and dopant reduces so that more triplet excitons can transfer energy to the dopant via DET, resulting in the energy loss of triplet excitons [57]. Consequently, an accurate control doping level in the preparation process is essential for efficient and stable energy transfer.

## 3. Doping-Free OLEDs Based on Interface Exciplex

As for reaching high-performance OLEDs based on exciplex, effective exciplex systems are essential [58,59,60]. Meanwhile, bulk exciplex has been attested to be a useful emitter and host for dyes that relies on an efficient and balanced charge transportation that can be achieved by employing donor and acceptor materials with high charge mobilities [61,62,63,64,65,66], whereas multi-doping technology and precise control of doping concentration aggravate manufacturing complexity [67,68,69,70,71]. Compared with bulk exciplex-based devices, interface exciplex-based devices can be easily achieved using doping-free technology. In this case, the ultrathin phosphorescent layer sandwiched between the donor and acceptor layers has been widely used to further simplify the fabrication process of OLEDs based on interface exciplex.

### 3.1. Doping-Free Monochrome OLEDs

**Figure 2:**
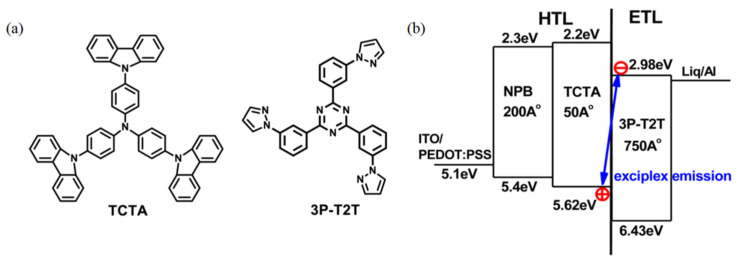
(**a**) Diagram of the TCTA and 3P-T2T molecular structures. (**b**) Diagram of the device structure with TCTA/3P-T2T exciplex emission [76].

Monochromatic OLEDs can be achieved by individual interface exciplex or an additional emitter. Although it was mentioned in earlier reports that exciplex formations are unfavorable for the efficiency of devices [72], the EQEs of recently reported OLEDs based on exciplex emission exceeded 5% due to the excellent TADF property of exciplexes [73,74,75]. Hung et al. first reported a simplified yellow exciplex OLED in 2013. As shown in Figure 2a, by employing 4,4′,4″-tri(N-carbazolyl)triphenylamine (TCTA) and 2,4,6-tris(3-(1H-pyrazol-1-yl)phenyl)-1,3,5-triazine (3P-T2T) as donor and acceptor, respectively, a bilayer structure EML is established, as shown in Figure 2b. Meanwhile, TCTA and 3P-T2T act as a hole transport layer (HTL) and electron transport layer (ETL), respectively [76]. Owing to the excellent charge transport mobilities of the two materials, charge carriers of holes and electrons can reach the donor/acceptor interface without limitation, leading to efficient exciton recombination [77]. The resulting device achieves a maximum CE, PE, and EQE of 22.5 cd/A, 23.6 lm/W, and 7.7%, respectively, along with a low turn-on voltage of 2 V. Because exciplex excitons are confined around the interface of the bilayer because of the relative higher T_1_s of the donor and acceptor, efficient triplet exciton harvest can occur via the RICS process, which is the principal cause of the high performance of the device. This work provides a simplified model of OLEDs based on interface exciplex, which suggests that the interface exciplex could be formed by the befitting combination of donor-acceptor pairs.

Furthermore, combining interface exciplex with ultrathin layer technology is another promising approach to achieving doping-free OLED [78]. By using complete energy transfer processes, which depend on the high overlapping between the absorption spectra of dyes and the exciplexes emission spectra, efficient utilization of excitons can be anticipated in ultrathin-layer-based devices [79,80].

**Figure 3:**
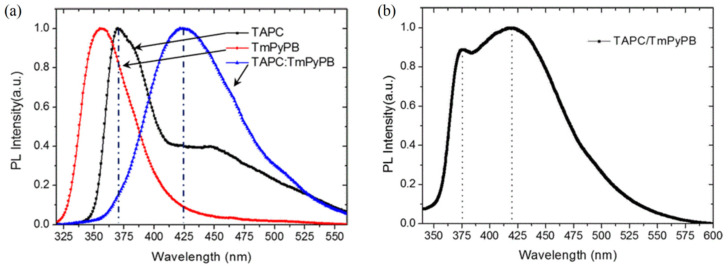
(**a**) The PL spectra of the TAPC, TmPyPB, and co-doped film. (**b**) The PL spectra of the TAPC/TmPyPB film [81].

In 2017, Xu et al. developed a green-emitting phosphorescent OLED based on an ultrathin emission layer (UEML) of 0.8 nm thick Bis[2-(2-pyridinyl-N)phenyl-C](acetylacetonato)iridium(III) (Ir(ppy)_2_acac). By inserting the UMEL into the interface of 1,1-Bis[(di-4-tolylamino)phenyl]cyclohexane (TAPC) and 1,3,5-tri(p-pyrid-3-ylphenyl)benzene (TmPyPB), a maximum EQE of 17.97% and CE of 66.2 cd/A were shown in the device. The PL spectra of constituent molecules and exciplex are shown in Figure 3. By further introducing a charge-generating unit (CGU) of Bathophenanthroline (Bphen): LiNH_2_/HAT-CN, a highly efficient green OLED based on the tandem structure can be constructed, achieving an increased CE of 135.74 cd/A and EQE of 36.85%. The superior performance can be attributed to the following: (i) exciplex formed at the TAPC/TmPyPB interface can transfer energy to the ultrathin layer efficiently because the PL spectrum of exciplex overlaps well with the absorption spectrum of the ultrathin layer; (ii) thanks to the higher T_1_s of TAPC and TmPyPB compared to exciplex, the reverse energy transfer from exciplex to the constructed molecules is well blocked, resulting in a sparkling green emission [81]. This OLED achieves efficient exciton harvesting by employing an interface exciplex and a tandem structure, which offers a basis for realizing efficient and simple OLEDs.

### 3.2. Doping-Free WOLEDs

Generally, interface-exciplex WOLEDs with a doping-free structure can be achieved by employing complementary emissions (blue and orange) or trichromatic emissions (blue, green, red) [82,83]. It is well-known that the health lighting source should have a high-color rendering index (CRI), which is easily improved by introducing an interface exciplex emission due to its broad spectrum composition [84,85].

**Figure 4:**
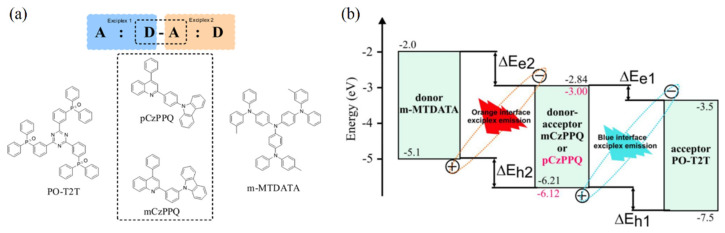
(**a**) Diagram of the molecular structures of PO-T2T, pCzPPQ, mCzPPQ, and m-MTDATA. (**b**) The sandwich structure of m-MTDATA/mCzPPQ or pCzPPQ/PO-T2T [86].

Sych et al. fabricated an A/D–A/D type WOLEDs by using doping-free technology with sandwich structures of m-MTDATA/pCzPPQ or mCzPPQ/PO-T2T, where m-MTDATA is 4,4′,4″-tris[3-methylphenyl(phenyl)amino]triphenylamine, pCzPPQ is 9-(4-(4-Phenylquinolin-2-yl)phenyl)-9H-carbazole, mCzPPQ is 9-(3-(4-Phenylquinolin-2-yl)phenyl)-9H-carbazole, and PO-T2T is 2,4,6-tris[3-(diphenylphosphinyl)phenyl]-1,3,5-triazine [86]. Meanwhile, D–A-type materials of pCzPPQ and mCzPPQ, which are the isomers of 9-phenylcarbazole and quinolone, are synthesized by Friedlander condensation and then the Ullmann cross-coupling reaction, as shown in Figure 4a. Therefore, blue and orange emissions can be formed at the dual interfaces of m-MTDATA/pCzPPQ (mCzPPQ) and pCzPPQ (mCzPPQ)/PO-T2T without extra donor layers, as shown in Figure 4b. Furthermore, color temperature can be easily recorded by modifying the thickness of the D–A functional system. The optimized all-exciplex-based device achieves a maximum EQE of 3.15% and a high CRI of 76. Recently, Wei et al. adopted a novel approach to construct an all-exciplex-based WOLEDs where white emission were formed from the same donor interface by parallelly depositing acceptor materials of TPBi and PO-T2T on the same donor layer of TAPC in a vertical direction. The device contains a unique interface exciplex architecture of TAPC (40 nm)/$\begin{array}{l} \mathrm{TBPi}\;(10\;\mathrm{nm})\\ \text{PO-T2T}\;(10\;\mathrm{nm}) \end{array}$, achieving a maximum CE of 3.17 cd/A with a high CRI of 71. By using this technology, simplified WOLEDs can be constructed by exciplex emission without elaborate molecule design engineering, leading to a reduced preparation complexity of WOLEDs [87].

The further improvements of efficiency of doping-free WOLEDs can be reached by introducing energy transfer processes from exciplexes to additional emission layers [88]. By employing high energy-state interface exciplex of 26DCzPPy/PO-T2T, Ying et al. realized high-efficiency doping-free WOLEDs using UEML. The main architectures of those WOLEDs are Ir(tptpy)_2_acac (0.1 nm)/26DczPPy (x nm)/FIrPic (0.3 nm)/PO-T2T, where Ir(tptpy)_2_acac is Iridium(III) bis(4-(4-t-butyl-phenyl)thieno[3,2-c]pyridinato-N,C2)acetylacetonate, bipolar 26DCzPPy is 2,6-bis(3-(carbazol-9-yl)phenyl)pyridine, and FIrPic is Iridium(III) bis[(4,6-difluorophenyl)-pyridinato-N,C2′]picolinate[89]. Under the scenario without the 26DczPPy layer, a weak blue emission and an intense orange emission will be generated from the FIrPic and Ir(tptpy)_2_acac, respectively, because the Ir(tptpy)_2_acac harvests most of the excitons of the low energy-state TCTA/PO-T2T interface exciplex. As the thickness of 26DCzPPy increases to 2 nm, the TCTA/PO-T2T exciplex formation is inhibited by the high energy-state 26DCzPPy/PO-T2T interface exciplex. The excitons energy of exciplex can be effectively transferred to Ir(tptpy)_2_acac and FIrPic, resulting in an EQE of 19.7%. Furthermore, devices achieve the correlated color temperature (CCT) with an adjustable range of 2878~9895 K by changing the thickness of the 26DCzPPy layer, which suggests that the energy transfer from interface exciplex to EMLs can be easily controlled.

However, full phosphorescent emitters always suffer from serious efficiency roll-off, although high efficiencies can be realized [90]. Fluorescence/phosphorescence hybrid WOLEDs have been proven to be a qualified approach to solve the problem. By introducing blue interface exciplex emission, Liu’s group realized doping-free WOLED without using additional blue emitters [91]. The m-MTDATA/3-(4-Biphenyl)-4-phenyl-5-tert-butylphenyl-1,2,4-triazole (TAZ) exciplex generates blue emissions and can excites orange phosphor of bis(2-(9,9-diethyl-9H-fluoren-2-yl)-1-phenyl-1Hbenzoimidazole-N,C3)iridium (acetylacetonate) [(fbi)_2_Ir(acac)] due to the higher T_1_. The energy transfer process from exciplex to the (fbi)_2_Ir(acac) can be adjusted by controlling the thickness of m-MTDATA in the m-MTDATA/TAZ interface exciplex forming system, and the balance between orange and blue emissions is achieved with 6 nm thick m-MTDATA. The resulting device achieves CRI of 81, which is one of the highest CRIs of the WOLEDs based on a single-emitter structure. By replacing (fbi)_2_Ir(acac) with the yellow phosphor of Ir(dmppy)_2_(dpp), the device achieves an EQE of 9.9%. Moreover, three-color WOLED is constructed by further employing additional red phosphor, achieving CRI and CCT of 85 and 2376 K, respectively.

**Figure 5:**
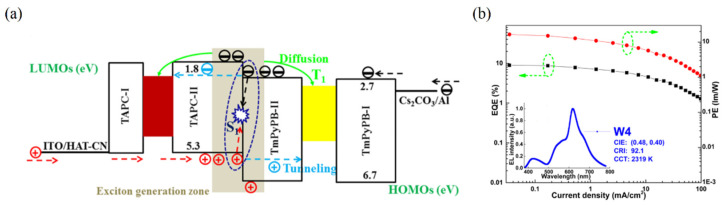
(**a**) Diagram of the device structure with exciplex/electroplex system. (**b**) The EQE and PE of the three-color WOLED with Exciplex/electroplex system [92].

Furthermore, the same group proposed another strategy to fabricate hybrid WOLEDs constructed by an exciplex/electroplex system and ultrathin emission layers [92]. The blue emission is generated by the combination of exciplex and electroplex, leading to broad EL spectra. The TAPC/TmPyPB interface exciplex and electroplex show peaks of 425 nm and 468 nm simultaneously under electric excitation. Therefore, a broader peak width at half maximum of the blue emissions can be furnished. As shown in Figure 5a, blue emissions come from singlets of the exciplex/electroplex system due to the short diffusion ratio of singlets. Meanwhile, red and yellow emissions can be attributed to multiple factors: (i) the emission of the exciplex/electroplex system itself; (ii) the diffusion processes of triplet excitons from the exciplex/electroplex system to EMLs; (iii) direct charge recombination due to the charge tunneling effect. Consequently, the hybrid WOLED obtains high efficiency by manipulating the exciplex/electroplex system, achieving EQE and CRI of 15.1% and 92.1, respectively (shown in Figure 5b).

**Figure 6:**
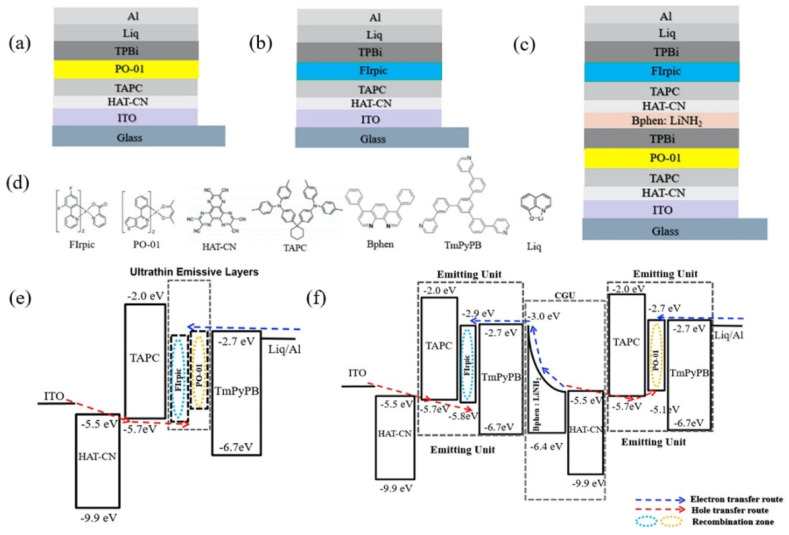
(**a**) Device structure of yellow OLED based on the UEML of PO−01. (**b**) Device structure of blue OLED based on the UEML of FIrPic. (**c**) Device structure of the tandem WOLED. (**d**) Diagram of molecular structures. (**e**) Schematic diagram of carrier transport in the yellow and blue monochrome OLEDs. (**f**) Schematic diagram of carrier transport in the tandem WOLED [93].

**Figure 7:**
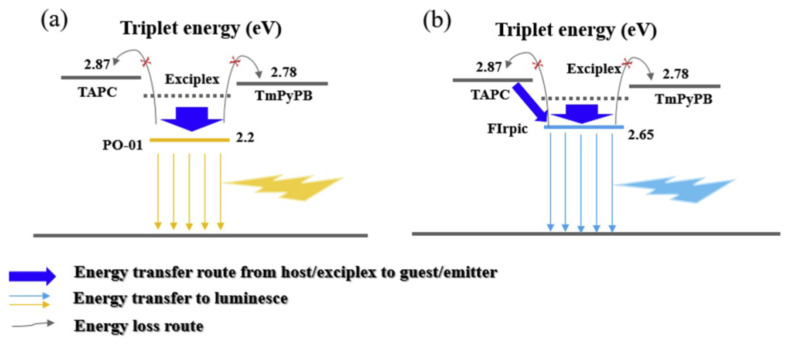
Energy transfer process in yellow and blue emission unit [93].

By combining ultrathin emission layers with interface exciplex, Xu et al. developed tandem WOLEDs, as shown in Figure 6 [93]. The yellow emission is generated by employing the phosphor of (acetylacetonato)bis[2-(thieno[3,2-c]pyridin-4-yl) phenyl]iridium(III) (PO-01) as an ultrathin emission layer to harvest the singlet and triplet excitons formed by TAPC/TmPyPB exciplex, and the reverse energy transfer from PO-01 to exciplex will be blocked because the T_1_ of PO-01 is lower than that of exciplex. Similarly, this mechanism is also applied to the blue emission unit, as shown in Figure 7. By connecting two emission units with the CGU of Bphen: LiNH_2_/HAT-CN, enhanced injections of electrons and holes into the emission units are achieved, with an EQE and CE of 18.59% and 41.5 cd/A, respectively, along with the turn-on voltage of 6.89 V.

## 4. Doped OLEDs Based on Interface Exciplex

The doping concentration is a significant factor determining doped device efficiency [94,95]. Within a reasonable concentration range, the device efficiency increases with the increase of the doping concentration [57]. However, it should be noted that a severe efficiency decline occurs under high doping concentrations owing to the increased possibility of direct electron-hole recombination on the dopant, which can be well inhibited by introducing an interface exciplex system. Thanks to the TADF properties of exciplex, the DET from interface exciplex to dopant will be inhibited, leading to weakening triplet exciton loss in the host–guest system.

### 4.1. Doped Monochrome OLEDs

Understanding exciton behavior is beneficial for manipulating excitons due to the different energy transfer paths between singlet and triplet excitons. For instance, the FRET from S_1_ of exciplex to dopant can be enhanced by the RISC process, weakening the DET between triplet excitons by augmenting the energy transfer distance; this means excitons become easy to manipulate by using interface exciplex. In the second half of this section, we will introduce several novel methods, including ternary exciplexes and applications of phosphor/TADF sensitization effects in interface exciplex devices

Kim et al. fabricated green-emitting phosphorescent OLEDs by doping fac-tris(2-phenyl-pyridine)iridium [Ir(ppy)_3_] into the donor layer of an N,N′-dicarbazolyl-4-4′-biphenyl (CBP)/bis-4,6-(3,5-di-3-pyridylphenyl)-2-methylpyrimidine (B3PYMPM) interface exciplex system [56]. The device efficiency increased with the doping concentration of Ir(ppy)_3_, which can be ascribed to the enhanced energy transfer efficiency from CBP/B3PYMPM exciplex to the Ir(ppy)_3_. The maximum EQE of the device was 20.1% with an Ir(ppy)_3_ doping concentration of 6%. By inserting an undoped CBP layer between the emission layer and the acceptor layer, the efficiency reduces as the thickness of the pure CBP layer rises, suggesting that the exciplex triplet excitons transfer is hindered, which can be explained by the fact that triplet exciton transferring is a short-range process.

By comparing the performance between an interface exciplex device and a bulk-exciplex device, Wang et al. drew the following conclusions: (i) the interface exciplex-based device shows a turn-on voltage of 2.36 V, which is lower than that of the bulk exciplex-based device because of the barrier-free electron-hole recombination; (ii) the interface exciplex-based device reaches a high PE of 97.2 lm/W, which is almost three times than that of the bulk exciplex-based device due to the elimination of charge traps of dye [96]. By employing a homemade material of 9-(4′-(4,6-diphenyl1,3,5-triazin-2-yl)-[1,1′-biphenyl]-3-yl)-9H-carbazole (o-DTPPC) as an acceptor to form TADF exciplex with TAPC, Duan et al. constructed a red-emitting phosphorescent OLED by interface exciplex architecture of TAPC/o-DTPPC: Ir(mphmq)_2_tmd, where Ir(mphmq)_2_tmd is (bis(4-methyl-2-(3,5-dimethylphenyl)quinoline))Ir(III)(tetramethylheptadionate) [53]. Owing to the small Δ*E_ST_*, the up-conversion from the triplet excitons of TAPC/o-DTPPC exciplex to singlet excitons via RISC can occur, leading to the enhanced FRET process from exciplex to red phosphor of Ir(mphmq)_2_tmd. Therefore, an efficient phosphorescent OLED is realized, achieving an EQE of 21.01%.

Generally, the unrecombined holes accumulated at the donor/acceptor interface could be a source of the degradation of the device [97]. By using a 5 nm thick emission layer, Colella et al. fabricated a red OLED by interface exciplex architecture of 26DCzPPy: 4% Ir(dmpq)_2_acac (5 nm)/PO-T2T, where Ir(dmpq)_2_acac is bis(2-(3,5-dimethylphenyl)quinoline-C2,N′)(acetylacetonato)-iridium(III), achieving a maximum EQE of 28.6%, along with increased lifetime [98]. The key reason for the superior performance is that the carrier recombination region is restrained to the 5 nm thick EML due to efficient Förster and Dexter energy transfer, resulting in a reduced concentration of excitons piled up at the heterojunction. By comparison, the devices without TADF systems exhibit declined performances and additional emissions from host materials at high voltage, which can be attributed to the broader exciton recombination regions in those devices.

In the course of application studies of OLEDs, reaching high-efficiency blue phosphorescent OLEDs with low driving voltages is still a challenge because it is hard to develop host materials with low singlet energy and high triplet energy [99,100,101,102,103]. Kido et al. demonstrated high performance blue-emitting phosphorescent OLEDs by doping FIrPic into the acceptor of 5′,5′′′′-sulfonyl-di-1,1′:3′,1″-terphenyl (BTPS), giving a PE of 50.1 lm/W and turn-on voltage of 2.5 V [77]. The excitons behavior is also studied by inserting an undoped BTPS layer (0~10 nm) into the TAPC/BTPS interface. The singlet excitons of TAPC/BTPS exciplex can be harvested by FIrPic via FRET when the thickness of the undoped BTPS is less than 5 nm, which is the main reason for high efficiency. In contrast, as the thickness of undoped BTPS increases to 10 nm, the device shows an efficiency decline, owing to the decrease of FRET efficiency.

Full fluorescent OLEDs are reported for pursuing low-cost and high-stability OLEDs [40,104]. However, fluorescent OLEDs based on conventional hosts always suffer from low EQEs owing to efficient DET from the T_1_ of hosts to the T_1_ of fluorophores [105,106]. The employment of interface exciplex is proposed to break this bottleneck because the DET from exciplex to fluorescent dopant is expected to be restricted due to the TADF properties of the interface exciplex. Moreover, direct exciton recombination on fluorescent dopants could be suppressed in interface exciplex systems, leading to decreased non-radiative transitions of triplet excitons.

Zhao et al. designed a fluorescent OLED by doping (5,6,11,12)-tetraphenyl-naphthacene (rubrene) into the interface exciplex system [55]. The absorption spectrum of rubrene overlaps well with the emission spectrum of the TCTA/3P-T2T exciplex, leading to an enhanced FRET process from exciplex to rubrene. Thanks to the TADF properties of the TCTA/3P-T2T interface exciplex, an efficient fluorescent OLED is realized, achieving an EQE of 8.1%. It is noteworthy that the doping concentration of rubrene in the device is as low as 1.5%. Thus, the DET process from triplet excitons of exciplex to triplet excitons of rubrene is inhibited, significantly reducing the waste of triplet excitons. By doping TADF polymer emitter PAPTC into the donor layer, Lin et al. developed a green-emitting OLED with the interface exciplex of TAPC/TmPyPB, obtaining a higher PL quantum yield than pure PAPTC, which can be attributed to the restrained aggregation effects of PAPTC polymer chains because the doping into TAPC provides a broad distribution area for PAPTC. Furthermore, thanks to the high *k_RISC_* of 1.48 × 10^7^ s^−1^ of the TAPC: PAPTC/TmPyPB exciplex system, a high-efficiency polymer electroluminescence OLED is constructed, achieving an EQE of 14.7% [107].

**Figure 8:**
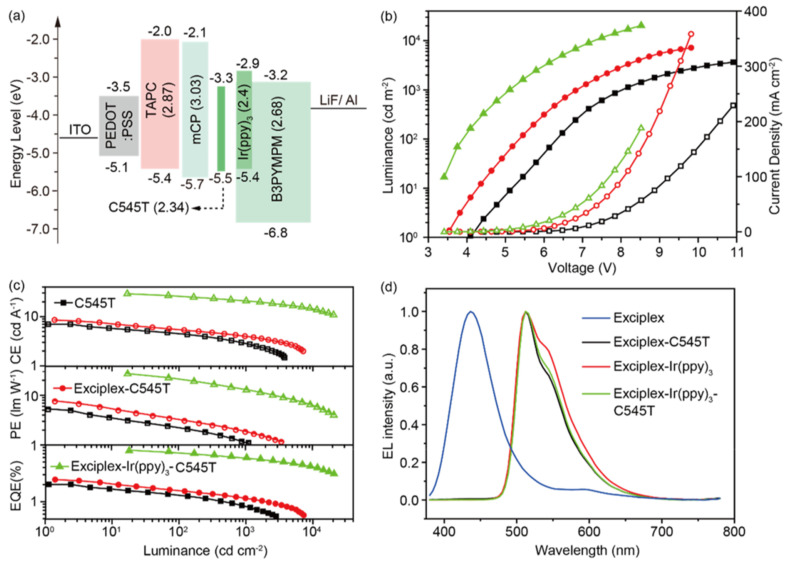
(**a**) Energy levels of materials used in fluorescent OLED. (**b**) The luminance−voltage−current density curves. (**c**) The efficiencies-luminance curves. (**d**) The EL spectra of OLEDs measured at 6 V [114].

**Figure 9:**
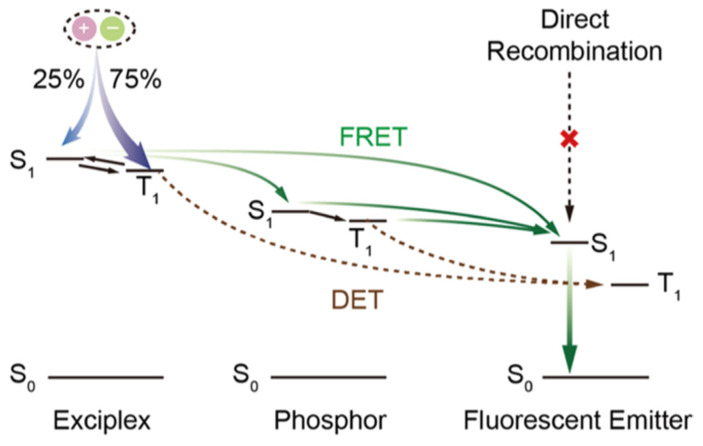
Diagram of energy transfer process in fluorescent OLED [114].

After deep and extensive research into doping technology [108,109,110,111,112,113], various novel strategies have been proposed to further improve the performances of interface exciplex-based devices. By adopting the strategy of synergistic sensitization using phosphor and interface exciplex, Chen et al. developed a green-emitting fluorescent OLED based on the ultrathin fluorescent emission layer, as shown in Figure 8. The 2,3,6,7-tetrahydro-1,1,7,7,-tetramethyl-1H,5H,11H-10-(2-benzothiazolyl)quinolizino-[9,9a,1gh]coumarin (C545T) can harvests excitons by two channels: Firstly, the N,N′-dicarbazolyl-3,5-benzene (mCP)/B3PYMPM exciplex possesses TADF properties, leading to the enhanced FRET process from exciplex to C545T. On the other hand, the exciplex excitons can be harvested by Ir(ppy)_3_, then the singlet and triplet excitons of Ir(ppy)_3_ transfer energy to C545T via FRET, as shown in Figure 9, which is efficient due to the approximate T_1_ between Ir(ppy)_3_ (2.4 eV) and C545T (2.34 eV). Moreover, the DET process from exciplex to C545T is well eliminated by employing Ir(ppy)_3_ as a sensitizer. Because the exciton utilization of exciplex is improved due to multiple efficient FRET processes, an efficient fluorescent device is constructed, achieving an EQE of 8.1% [114].

**Figure 10:**
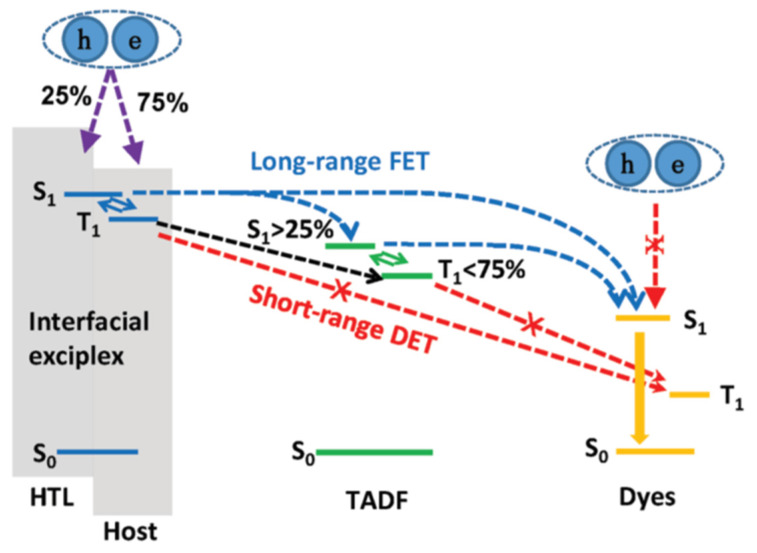
Diagram of energy transfer mechanisms of device with TADF sensitizer [115].

Duan et al. developed an efficient green-emitting fluorescent OLED by employing TADF sensitizer [115]. By using electronic inert terminal substituents to shield the electronically active core of fluorophore of N9,N9,N10,N10-tetraphenylanthracene-9,10-diamine (PAD), the N9,N9,N10,N10-tetrakis(4-(2-phenylpropan-2-yl)phenyl)anthracene-9,10-diamine (PhtBuPAD) was synthesized. The PhtBuPAD exhibits reduced orbital overlaps with neighboring molecules owing to the large steric effect of inert units, which can inhibit the impact of the DET process. By introducing the TCTA/3-(3-(4,6-diphenyl-1,3,5-triazin-2-yl)- phenyl)-9-phenyl-9H-carbazole (PhCzTrz) interface exciplex with TADF property, the adverse direct electron-hole recombination on 10,10′-(sulfonylbis(4,1-phenylene))bis(10H-phenoxazine) (PXZ-DPS) is suppressed. Meanwhile, triplet excitons that are not used by the RISC process of the exciplex host can transfer energy to PXZ-DPS and further upconvert it into singlet excitons via RISC, leading to efficient FRET from TADF sensitizer to PhtBuPAD, as shown in Figure 10. Therefore, PhtBuPAD harvests more singlet excitons via two-fold RISC processes due to the TADF properties of exciplex and PXZ-DPS. As a result, a very efficient device is constructed, achieving an EQE of 24%, along with an extremely low efficiency roll-off.

**Figure 11:**
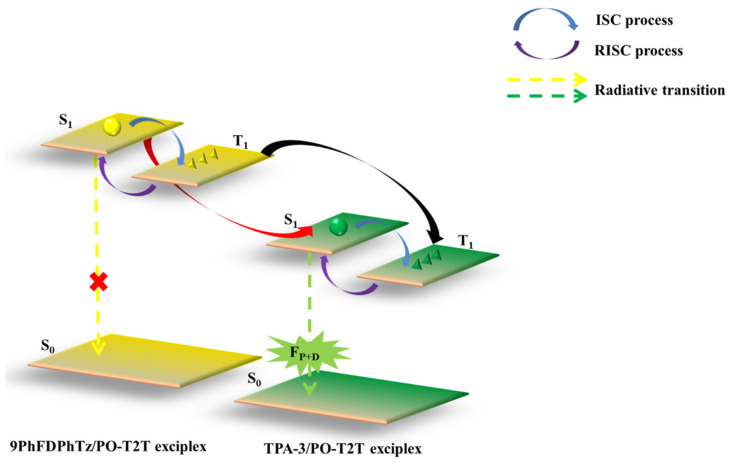
Diagram of energy transfer process of ternary exciplexes.

As discussed above, increasing the RISC channel has proven to be an efficient method to improve the performance of exciplex-based OLEDs [116]. By employing 9-(4-(9-(4,6-diphenyl-1,3,5-triazin-2-yl)-9H-fluoren-9-yl)phenyl)-9H-carbazole (9PhFDPhTz) and the homemade small molecule material of 4-(9-(perfluoropyridin-4-yl)-9H-fluoren-9-yl)-N,N-diphenylaniline (TPA-3) as donors to form ternary exciplexes with the acceptor of PO-T2T, Zhang et al. fabricated a green-emitting OLED by employing multiple RISC technologies [117]. It should be noted that the 9PhFDPhTz/PO-T2T exciplex in this device does not emit light but only acts as an assistant to enhance the RISC process of the ternary exciplexes. Figure 11 shows that the excitons energy transfer from 9PhFDPhTz/PO-T2T to TPA-3/PO-T2T can occur because the 9PhFDPhTz/PO-T2T exciplex exhibits higher T_1_ than that of the TPA-3/PO-T2T exciplex. This energy transfer will be more effective because the solution process method induces stronger intermolecular interactions. As a result, an efficient exciplexes-based OLED is developed, achieving an EQE of 24%, which is one of the best values among exciplex-emission devices. Furthermore, the ternary exciplexes device shows a lower efficiency roll-off than the binary TPA-3/PO-T2T exciplex-based device because of the additional RISC path.

Various OLEDs based on interface exciplex have been introduced in the above paragraphs. It is worth noting that the donor and acceptor molecules that form the interface exciplex are aggregated in a narrow interface area in the D/A structure, resulting in acute intermolecular interactions and a concentration-quenching effect of triplet excitons [118,119]. Recently, a novel strategy that was constructed by employing spatially separated electron-hole pairs has attracted the attention of researchers, and distributes excitons in a broad region to reduce exciton concentration. Adachi et al. first proposed the donor-spacer-acceptor (D-S-A) structure with a spatially separated donor and acceptor [120]. In a device with a substructure of m-MTDATA/3,3-di(9H-carbazol-9-yl)biphenyl(mCBP)/2,4,6-tris(biphenyl-3-yl)-1,3,5-triazine (T2T), they designed a bipolar material of mCBP that acts as the electron acceptor and electron donor simultaneously to form exciplexes with m-MTDATA and T2T, respectively. Because the molecules of donor and acceptor are directly excited, a charge transfer between D^δ+^/S^δ−^/A and D/S^δ+^/A^δ−^ occurs to form exciplexes of D^δ+^/S/A^δ−^ by cascade electron transfer between the three molecules. Here, the up-conversion of ^3^(D^δ+^A^δ−^)* into ^1^(D^δ+^A^δ−^)* occurs via RISC due to the TADF properties of exciplex. It is noteworthy that both the T_1_ of m-MTDATA and T2T is lower than that of mCBP, which prevents the energy transfer process between exciplex and mCBP. By changing the thickness of mCBP, the exciton energy and radiative decay lifetime of exciplex can be fine-tuned. Furthermore, long-range coupling CT excited states under electrical excitation are examined, and can be explained by the following expression: D^+^ + A^−^ → ^1^(D^δ+^A^δ−^)* → *hν*_ex_ + D + A. Finally, the feasibility of energy transfer from exciplex to fluorescent dopant is confirmed without direct charge injection into the dopant.

In 2018, Su et al. reported efficient fluorescent OLEDs with high efficiency by adopting a spatially separated exciplex system [121]. Devices with 1–4 nm-thick spacers of mCP showed a higher brightness, increased efficiencies, and restrained efficiency roll-offs than the device without spacer layers. By further doping 1% DBP into TAPC, the device with 3 nm-thick mCP reached the highest EQE of 14.8%, which can be ascribed to the following: (i) the spacer layer provides a broad exciton distribution region that inhibits exciton quenching; (ii) the direct charge trapping is suppressed due to separated hole-electron pairs; (iii) the FRET efficiency from exciplex to DBP is enhanced by using a separated exciplex system. Pu et al. further studied the mechanism of long-range coupling electron-hole pairs. What is impressive is that, although the thickness of the spacer layer of (9,10-bis(3,5-dimethoxyphenyl)anthracene) (DMA) increases to 70 nm in the device they studied, the weak long-range coupling CT excited states can still exist, which is beyond expectations and worth further investigate [122]. The EL performances of monochrome OLEDs based on interface exciplex are summarized in Table 1.

### 4.2. Doped WOLEDs

Combining interface exciplex with doping technology, doped WOLEDs can simultaneously reach high efficiencies and low driving voltages while maintaining simple device structures. The EL performances of WOLEDs based on interface exciplex are summarized in Table 2.

**Figure 12:**
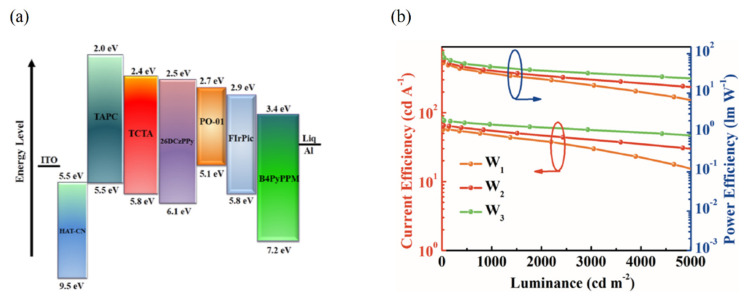
(**a**) Diagram of energy levels of the materials used in single−emission−layer WOLED. (**b**) The current efficiency−luminance−power efficiency curves of WOLEDs [123].

Recently, our group developed highly efficient phosphorescence WOLEDs by co-doping a complementary emitter into an acceptor of 4,6-bis(3,5-di(pyridin-4-yl)phenyl)-2-phenylpyrimidine (B4PyPPM) (shown in Figure 12a) [123]. A novel interfacial exciplex system 26DCzPPy/B4PyPPM was used to build white OLEDs based on the single-emission-layer structure, achieving efficiencies of 101.9 lm/W and 81.1 cd/A, along with a low turn-on voltage of 2.4 V, which is one of the best values in WOLEDs based on simplified architecture, as shown in Figure 12b. The enhanced performance could be attributed to the reduced energy loss of triplet excitons in the host through efficient FRET between the exciplex and the dopants, and the DET energy transfer process between the blue and orange dopants is suppressed due to the low doping concentration of orange dye. Additionally, a single-emission-layer white OLED based on an interface exciplex system only requires the doping of three materials simultaneously, with no need for high-performance host materials, providing a promising method to design simplified white OLEDs with high performance for commercial solid-state lighting and display.

**Figure 13:**
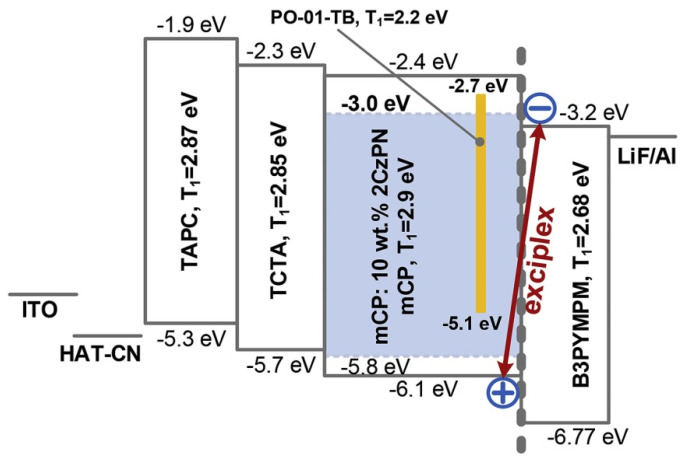
Schematic diagram of the energy level of hybrid WOLED [124].

Gao et al. reported a high-efficiency and color-stable hybrid WOLED, as shown in Figure 13 [124]. By investigating PL and charge transport properties, the author demonstrates direct electron-hole recombination on a blue TADF fluorophore of 4,5-bis(carbazol-9-yl)-1,2-dicyanobenzene (2CzPN), which was well restrained, so that the blue emission from 2CzPN was mainly generated from the energy transfer from the mCP/B3PYMPM interface exciplex. On the other hand, the yellow phosphor Iridium(III) bis(4-(4-tert-butylphenyl) thieno[3,2-c]pyridinato-N,C2′) acetylacetonate (PO-01-TB) inserted into mCP as the complementary ultrathin emission layer was excited by the following two main paths: (i) the HOMO level of PO-01-TB is shallower than that of TCTA, which means that PO-01-TB is a trapping site for holes, causing direct electron-hole recombination on PO-01-TB; (ii) the T_1_ of 2CzPN is higher than that of PO-01-TB, leading to the DET from 2CzPN to PO-01-TB. Because the exciplex excitons can be efficiently utilized, a warm white device is realized with an EQE of 22.3%.

**Figure 14:**
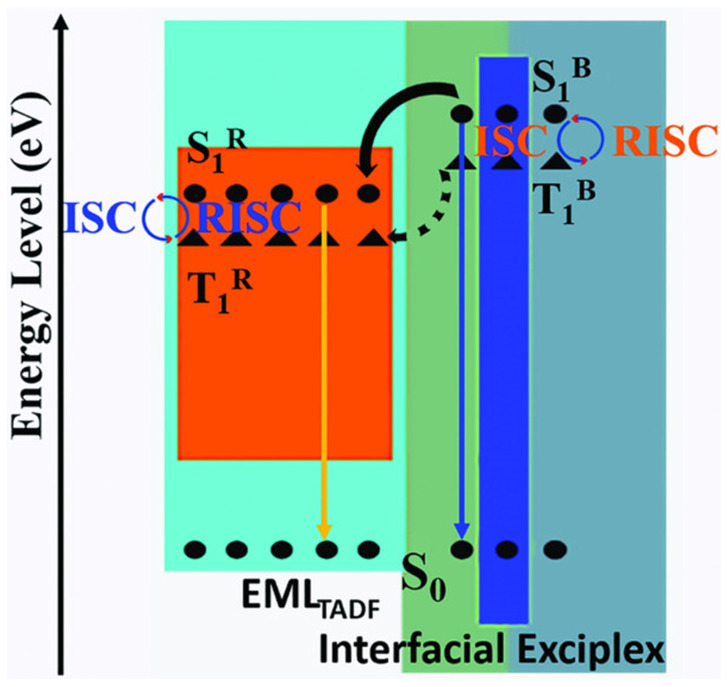
Diagram of energy transfer from CDBP/B4YPPM exciplex to orange-red emitter TXO-TPA [125].

**Figure 15:**
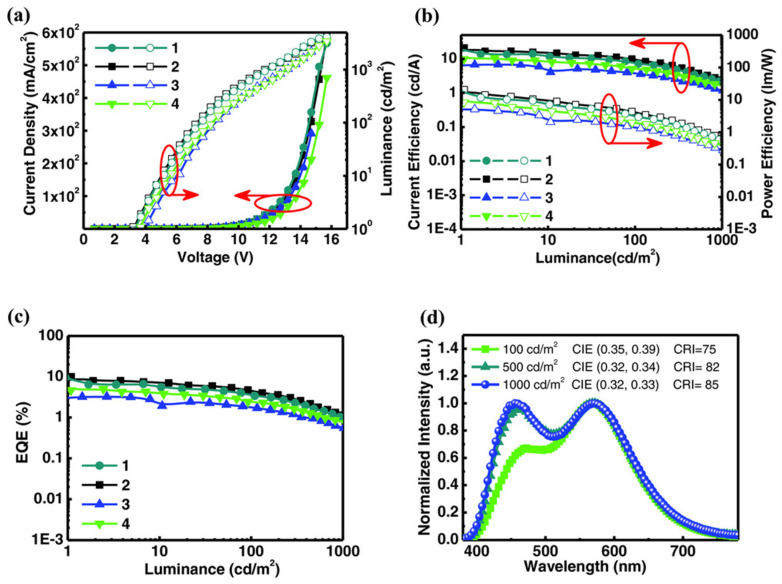
(**a**) current density-voltage-luminance characteristics of the WOLEDs with thicknesses of CDBP of 2.5, 3.5, 4.0, and 5.0 nm, corresponding to curves 1, 2, 3, and 4. (**b**) The current efficiency-luminance-power efficiency characteristics. (**c**) The EQE-luminance characteristics. (**d**) The EL spectra of the device with 3.5 nm-thick CDBP [125].

By manipulating excitons with CDBP/B4PyPPM interface exciplex, Wang et al. constructed color-stable solution-processed WOLED [125]. The triplet excitons can be well confined to the exciplex because of the relatively low T_1_ of 4,4′-bis(9-carbazolyl)-2,2′-dimethylbip-henyl (CDBP)/B4PyPPM exciplex, leading to efficient up-conversion from triplet excitons to singlet excitons via RISC to enhance blue emission. The orange-red emissions are generated from the TADF emitter of 2-[4-(diphenylamino) phenyl]-10,10-dioxide-9H-thioxanthen-9-one (TXO-TPA). The energy transfer process from exciplex to TXO-TPA is shown in Figure 14. Note that the CDBP/B4PyPPM exciplex and TXO-TAP have no direct contact because of the 3.5 nm-thick CDBP, resulting in the DET process from blue exciplex to TXO-TPA being well suppressed. Therefore, orange-red emission is enhanced by transferring energy from the blue TADF exciplex to TXO-TPA via FRET, which can be fine-tuned by changing the thickness of the CDBP. By contrast, bulk exciplex of the CDBP: B4PyPP-based device suffers from the shift of the exciton recombination region with increasing voltage or current density due to unfavorable exciton management, leading to unstable spectra. The continuous spectra superimposed by blue emission and orange-red emission spectrum covers the entire visible spectral region, achieving a CRI of 85 in the resulting device, as shown in Figure 15.

**Table 2:** Summary of EL performance of WOLEDs based on interface exciplex.

Devices	CIE (x, y)	CRI	Turn-on Voltage (V)	EQE_max_/PE_max_/CE_max_
				%/lm W^−1^/cd A^−1^
Ref [86]	(0.21, 0.56) ^a^	76	6.0	3.15/4.45/8.9
Ref [87]	(0.385, 0.401) ^b^	71	3.0	1.25/2.53/3.17
Ref [89]	(0.46, 0.46) ^b^	86	2.4	19.6/83.2/63.3
Ref [91]	–	85	–	11.3/23.5/20.9
Ref [92]	(0.48, 0.40) ^c^	92.1	2.85	15.1/28.2/26.9
Ref [93]	(0.36, 0.41) ^d^	–	6.89	18.59/18.92/41.5
Ref [123]	(0.47, 0.50) ^c^	–	2.4	23.1/101.9/81.1
Ref [124]	(0.44, 0.51) ^c^	–	2.6	22.3/79.2/65.9
Ref [125]	(0.32, 0.33) ^c^	85	3.2	10.02/20.71/21.10
Ref [126]	(0.40, 0.44) ^c^	–	6.8	18.8/19.3/53.8
Ref [127]	–	–	2.3	5/–/15
Ref [128]	(0.41, 0.40) ^e^	77	–	20.0/31.7/40.0
Ref [129]	(0.33, 0.39) ^c^	75.7	4.1	20.8/31.3/–

EQE_max_: The maximum external quantum efficiency. PE_max_: The maximum power efficiency. CE_max_: The maximum current efficiency. ^a^ Measured at 12 V. ^b^ Measured at 4 V. ^c^ Measured at 1000 cd m^−2^. ^d^ Measured at 83 cd m^−2^. ^e^ Measured at 5 V.

## 5. Conclusions and Outlook

In this review paper, the basic concepts and applications of interface exciplex are demonstrated. Interface exciplex is the charge transfer state formed by donor and acceptor molecules at the donor/acceptor interface, which can be used as an emitter or energy transfer host. The success of interface exciplex as an emitter relies on an efficient RISC process, which improves the fluorescence quantum yield. Therefore, the EQE of OLED based on exciplex emission can exceed 5%. Moreover, introducing the energy transfer process between exciplex and dopant is an effective approach to further improve the efficiency of interface exciplex-based devices, achieving low turn-on voltage and inhibiting direct electron-hole recombination on the dopant. However, the interface exciplex system is still faced with problems such as serious triplet exciton quenching owing to a narrow distribution area and unfavorable fluorescence quantum efficiency because of low RISC rate. Despite providing a broad exciton recombination zone by adopting the long-range coupling CT excited states strategy, the EQE of a device with a D/S/A structure is far below a device with D/A interface exciplex. For now, improving the efficiency of the RISC is the most feasible and practical solution to those problems. Therefore, further studies are necessary to investigate the radiative transition process in interface exciplex, and novel interface exciplex systems with a high-fluorescence quantum yield still need to be developed to accelerate the commercialization process of interface exciplex-based devices.

## Figures and Tables

**Table 1:** Summary of EL performance of monochrome OLEDs based on interface exciplex.

Devices	Turn-on Voltage (V)	EQE_max_ (%)	PE_max_ (lm W^−1^)	CE_max_ (cd A^−1^)	Emission Color
Ref [76]	2.0	7.7	–	–	Yellow
Ref [53]	4.35	21.01	17.95	31.36	Red
Ref [55]	2.37	8.1	22.6	25.3	Red
Ref [56]	–	20.1	–	–	Green
Ref [75]	2.66	24.4	47.4	40.8	Orange-red
Ref [77]	2.5	21.7	50.1	46.1	Blue
Ref [78]	3.6	14.3	37.4	40.5	Yellow
Ref [81]	5.76	36.85	59.88	135.74	Green
Ref [96]	2.36	25.2	97.2	74.3	Yellow
Ref [98]	4.0	28.6	–	–	Red
Ref [107]	2.5	14.9	50.1	–	Green
Ref [114]	3.4	8.1	25.7	27.9	Green
Ref [115]	2.55	24.0	71.4	–	Green
Ref [117]	3.1	24.0	61.4	78.2	Green
Ref [119]	2.86	26.9	92.8	84.5	Green

EQE_max_: The maximum external quantum efficiency. PE_max_: The maximum power efficiency. CE_max_: The maximum current efficiency.

## Data Availability

Not applicable.

## References

[1] Kalyani N.T., Dhoble S.J. (2012). Organic light emitting diodes: Energy saving lighting technology—A review. Renew. Sust. Energy Rev..

[2] Shi H., Deng L., Chen S., Xu Y., Zhou H., Cheng F., Li X., Wang L., Huang W. (2014). Flexible top-emitting warm-white organic light-emitting diodes with highly luminous performances and extremely stable chromaticity. Org. Electron..

[3] Deng L., Yang J., Zhan N., Yu T., Yu H., Chen S. (2019). High-performance solution-processed white organic light-emitting diodes based on silica-coated silver nanocubes. Opt. Lett..

[4] Eritt M., May C., Leo K., Toerker M., Radehaus C. (2010). OLED manufacturing for large area lighting applications. Thin Solid Films.

[5] Sasabe H., Kido J. (2013). Development of high performance OLEDs for general lighting. J. Mater. Chem. C.

[6] Kim J.Y., Joo C.W., Lee J., Woo J.-C., Oh J.-Y., Baek N.S., Chu H.Y., Lee J.-I. (2015). Save energy on OLED lighting by a simple yet powerful technique. RSC Adv..

[7] Baldo M.A., O’Brien D.F., You Y., Shoustikov A., Sibley S., Thompson M.E., Forrest S.R. (1998). Highly efficient phosphorescent emission fromorganic electroluminescent devices. Nature.

[8] Zhu H., Badia-Dominguez I., Shi B., Li Q., Wei P., Xing H., Ruiz Delgado M.C., Huang F. (2021). Cyclization-Promoted Ultralong Low-Temperature Phosphorescence via Boosting Intersystem Crossing. J. Am. Chem. Soc..

[9] Shafikov M.Z., Zaytsev A.V., Suleymanova A.F., Brandl F., Kowalczyk A., Gapinska M., Kowalski K., Kozhevnikov V.N., Czerwieniec R. (2020). Near Infrared Phosphorescent Dinuclear Ir(III) Complex Exhibiting Unusually Slow Intersystem Crossing and Dual Emissive Behavior. J. Phys. Chem. Lett..

[10] Zhang H., Guo Y., Wu Z., Wang Y., Sun Y., Feng X., Wang H., Zhao G. (2021). Unveiling the theoretical mechanism of purely organic room temperature phosphorescence emission and heteroatomic effects on singlet-triplet intersystem crossing for isopropylthioxanthone derivatives. J. Lumin..

[11] Sun Y., Liu B., Guo Y., Feng Z., Zhou G., Chen Z., Yang X. (2021). Triphenylamine-based trinuclear Pt(II) complexes for solution-processed OLEDs displaying efficient pure yellow and red emissions. Org. Electron..

[12] Uoyama H., Goushi K., Shizu K., Nomura H., Adachi C. (2012). Highly efficient organic light-emitting diodes from delayed fluorescence. Nature.

[13] Kim H.-B., Kim J.-J. (2019). Recent progress on exciplex-emitting OLEDs. J. Inf. Disp..

[14] Noda H., Nakanotani H., Adachi C. (2018). Excited state engineering for efficient reverse intersystem crossing. Sci. Adv..

[15] Endo A., Sato K., Yoshimura K., Kai T., Kawada A., Miyazaki H., Adachi C. (2011). Efficient up-conversion of triplet excitons into a singlet state and its application for organic light emitting diodes. Appl. Phys. Lett..

[16] dos Santos P.L., Dias F.B., Monkman A.P. (2016). Investigation of the Mechanisms Giving Rise to TADF in Exciplex States. J. Phys. Chem. C.

[17] Zhang L., Cai C., Li K.F., Tam H.L., Chan K.L., Cheah K.W. (2015). Efficient Organic Light-Emitting Diode through Triplet Exciton Reharvesting by Employing Blended Electron Donor and Acceptor as the Emissive Layer. ACS Appl. Mater. Interfaces.

[18] Sarma M., Wong K.T. (2018). Exciplex: An Intermolecular Charge-Transfer Approach for TADF. ACS Appl. Mater. Interfaces.

[19] Chen S., Wu Z., Zhao Y., Li C., Hou J., Liu S. (2005). Efficient organic light-emitting device from exciplex emission between 4,4′,4″-tris[3-methylphenyl(phenyl)amino]triphenylamine and 2,2′,2″-(1,3,5-benzenetriyl)tris-[1-phenyl-1H-benzimidazole]. Org. Electron..

[20] Fan C., Duan C., Wei Y., Ding D., Xu H., Huang W. (2015). Dibenzothiophene-Based Phosphine Oxide Host and Electron-Transporting Materials for Efficient Blue Thermally Activated Delayed Fluorescence Diodes through Compatibility Optimization. Chem. Mater..

[21] Liu B., Wang L., Tao H., Xu M., Zou J., Ning H., Peng J., Cao Y. (2017). Doping-free tandem white organic light-emitting diodes. Sci. Bull..

[22] Zhao J., Yuan S., Du X., Li W., Zheng C., Tao S., Zhang X. (2018). White OLEDs with an EQE of 21% at 5000 cd m^−2^ and Ultra High Color Stability Based on Exciplex Host. Adv. Opt. Mater..

[23] Sasabe H., Sato R., Suzuki K., Watanabe Y., Adachi C., Kaji H., Kido J. (2018). Ultrahigh Power Efficiency Thermally Activated Delayed Fluorescent OLEDs by the Strategic Use of Electron-Transport Materials. Adv. Opt. Mater..

[24] Ng T.W., Lo M.F., Fung M.K., Zhang W.J., Lee C.S. (2014). Charge-transfer complexes and their role in exciplex emission and near-infrared photovoltaics. Adv. Mater..

[25] He S.J., Wang D.K., Jiang N., Tse J.S., Lu Z.H. (2016). Tunable Excitonic Processes at Organic Heterojunctions. Adv. Mater..

[26] Liu X.K., Chen Z., Zheng C.J., Liu C.L., Lee C.S., Li F., Ou X.M., Zhang X.H. (2015). Prediction and design of efficient exciplex emitters for high-efficiency, thermally activated delayed-fluorescence organic light-emitting diodes. Adv. Mater..

[27] Nobuyasu R.S., Ren Z., Griffiths G.C., Batsanov A.S., Data P., Yan S., Monkman A.P., Bryce M.R., Dias F.B. (2016). Rational Design of TADF Polymers Using a Donor-Acceptor Monomer with Enhanced TADF Efficiency Induced by the Energy Alignment of Charge Transfer and Local Triplet Excited States. Adv. Opt. Mater..

[28] Li W., Pan Y., Xiao R., Peng Q., Zhang S., Ma D., Li F., Shen F., Wang Y., Yang B. (2014). Employing ∼100% Excitons in OLEDs by Utilizing a Fluorescent Molecule with Hybridized Local and Charge-Transfer Excited State. Adv. Funct. Mater..

[29] Lee J.-H., Shin H., Kim J.-M., Kim K.-H., Kim J.-J. (2017). Exciplex-Forming Co-Host-Based Red Phosphorescent Organic Light-Emitting Diodes with Long Operational Stability and High Efficiency. ACS Appl. Mater. Interfaces.

[30] Goushi K., Adachi C. (2012). Efficient organic light-emitting diodes through up-conversion from triplet to singlet excited states of exciplexes. Appl. Phys. Lett..

[31] Giro G., Cocchi M., Kalinowski J., Di Marco P., Fattori V. (2000). Multicomponent emission from organic light emitting diodes based on polymer dispersion of an aromatic diamine and an oxadiazole derivative. Chem. Phy. Lett..

[32] Zhang Q., Tsang D., Kuwabara H., Hatae Y., Li B., Takahashi T., Lee S.Y., Yasuda T., Adachi C. (2015). Nearly 100% internal quantum efficiency in undoped electroluminescent devices employing pure organic emitters. Adv. Mater..

[33] Angioni E., Chapran M., Ivaniuk K., Kostiv N., Cherpak V., Stakhira P., Lazauskas A., Tamulevičius S., Volyniuk D., Findlay N.J. (2016). A single emitting layer white OLED based on exciplex interface emission. J. Mater. Chem. C.

[34] Zhang T., Zhao B., Chu B., Li W., Su Z., Wang L., Wang J., Jin F., Yan X., Gao Y. (2015). Blue exciplex emission and its role as a host of phosphorescent emitter. Org. Electron..

[35] Jankus V., Chiang C.J., Dias F., Monkman A.P. (2013). Deep blue exciplex organic light-emitting diodes with enhanced efficiency; P-type or E-type triplet conversion to singlet excitons?. Adv. Mater..

[36] Zhao B., Zhang T., Chu B., Li W., Su Z., Luo Y., Li R., Yan X., Jin F., Gao Y. (2015). Highly efficient tandem full exciplex orange and warm white OLEDs based on thermally activated delayed fluorescence mechanism. Org. Electron..

[37] Yuan W., Hu D., Zhu M., Shi W., Shi C., Sun N., Tao Y. (2021). Simple peripheral modification for color tuning of thermally activated delayed fluorescence emitters in OLEDs. Dyes. Pigm..

[38] Kalinowski J. (2009). Excimers and exciplexes in organic electroluminescence. Mater. Sci. Poland.

[39] Baldo M.A., O’Brien D.F., Thompson M.E., Forrest S.R. (1999). Excitonic singlet-triplet ratio in a semiconducting organic thin film. Phys. Rev. B.

[40] Goushi K., Yoshida K., Sato K., Adachi C. (2012). Organic light-emitting diodes employing efficient reverse intersystem crossing for triplet-to-singlet state conversion. Nat. Photonics.

[41] Yuan P., Guo X., Qiao X., Yan D., Ma D. (2019). Improvement of the Electroluminescence Performance of Exciplex-Based OLEDs by Effective Utilization of Long-Range Coupled Electron–Hole Pairs. Adv. Optical Mater..

[42] Yang D., Kim J.-M., Huh J.-S., Kim J.-J., Hong J.-I. (2021). The effect of the electron-donor ability on the OLED efficiency of twisted donor-acceptor type emitters. Org. Electron..

[43] Graves D., Vygintas J.V., Dias F.B., Monkman A. (2014). Photophysical Investigation of the Thermally Activated Delayed Emission from Films of m-MTDATA:PBD Exciplex. Adv. Funct. Mater..

[44] Wang J., Chen J., Qiao X., Alshehri S.M., Ahamad T., Ma D. (2016). Simple-Structured Phosphorescent Warm White Organic Light-Emitting Diodes with High Power Efficiency and Low Efficiency Roll-off. ACS Appl. Mater. Interfaces.

[45] Al Attar H.A., Monkman A.P. (2016). Electric Field Induce Blue Shift and Intensity Enhancement in 2D Exciplex Organic Light Emitting Diodes; Controlling Electron-Hole Separation. Adv. Mater..

[46] Sun J.W., Lee J.H., Moon C.K., Kim K.H., Shin H., Kim J.J. (2014). A fluorescent organic light-emitting diode with 30% external quantum efficiency. Adv. Mater..

[47] Seino Y., Inomata S., Sasabe H., Pu Y.J., Kido J. (2016). High-Performance Green OLEDs Using Thermally Activated Delayed Fluorescence with a Power Efficiency of over 100 lm W^−1^. Adv. Mater..

[48] Wang Z., Wang C., Zhang H., Liu Z., Zhao B., Li W. (2019). The application of charge transfer host based exciplex and thermally activated delayed fluorescence materials in organic light-emitting diodes. Org. Electron..

[49] Zhao C., Yan D., Ahamad T., Alshehri S.M., Ma D. (2019). High efficiency and low roll-off hybrid white organic light emitting diodes by strategically introducing multi-ultrathin phosphorescent layers in blue exciplex emitter. J. Appl. Phys..

[50] Berenis D., Kreiza G., Juršėnas S., Kamarauskas E., Ruibys V., Bobrovas O., Adomėnas P., Kazlauskas K. (2020). Different RISC rates in benzoylpyridine-based TADF compounds and their implications for solution-processed OLEDs. Dyes. Pigm..

[51] Zysman-Colman E. (2020). Molecular designs offer fast exciton conversion. Nat. Photonics.

[52] Seino Y., Sasabe H., Pu Y.J., Kido J. (2014). High-performance blue phosphorescent OLEDs using energy transfer from exciplex. Adv. Mater..

[53] Song X., Zhang D., Huang T., Cai M., Duan L. (2018). Efficient red phosphorescent OLEDs based on the energy transfer from interface exciplex: The critical role of constituting molecules. Sci. China Chem..

[54] Xia Y., Liu Z., Li J., Fan C., Li G., Zhao B., Wu Y., Wang H., Guo K. (2020). TADF material with non-conjugated rigid donor for high-performance full-color phosphorescent OLEDs: Effects of triplet harvest and charge transport on efficiency. Org. Electron..

[55] Zhao B., Miao Y., Wang Z., Chen W., Wang K., Wang H., Hao Y., Xu B., Li W. (2016). Highly efficient orange fluorescent OLEDs based on the energy transfer from bilayer interface exciplex. Org. Electron..

[56] Park Y.-S., Jeong W.-I., Kim J.-J. (2011). Energy transfer from exciplexes to dopants and its effect on efficiency of organic light-emitting diodes. J. Appl. Phys..

[57] Zhang D., Cai M., Zhang Y., Zhang D., Duan L. (2015). Highly Efficient Simplified Single-Emitting-Layer Hybrid WOLEDs with Low Roll-off and Good Color Stability through Enhanced Forster Energy Transfer. ACS Appl. Mater. Interfaces.

[58] Yao B., Lin X., Zhang B., Wang H., Liu X., Xie Z. (2018). Power-efficient and solution-processed red phosphorescent organic light-emitting diodes by choosing combinations of small molecular materials to form a well-dispersed exciplex co-host. J. Mater. Chem. C.

[59] Baek H.J., Lee S.E., Lee H.W., Yun G.J., Park J., Kim W.Y., Kim Y.K. (2018). White Organic Light-Emitting Diodes Using Exciplex Emission with Multiple Emitting Layers. Phys. Status Solidi A.

[60] Lai S.L., Chan M.Y., Tong Q.X., Fung M.K., Wang P.F., Lee C.S., Lee S.T. (2008). Approaches for achieving highly efficient exciplex-based organic light-emitting devices. Appl. Phys. Lett..

[61] Park Y.-S., Lee S., Kim K.-H., Kim S.-Y., Lee J.-H., Kim J.-J. (2013). Exciplex-Forming Co-host for Organic Light-Emitting Diodes with Ultimate Efficiency. Adv. Funct. Mater..

[62] Hung W.Y., Chiang P.Y., Lin S.W., Tang W.C., Chen Y.T., Liu S.H., Chou P.T., Hung Y.T., Wong K.T. (2016). Balance the Carrier Mobility to Achieve High Performance Exciplex OLED Using a Triazine-Based Acceptor. ACS Appl. Mater. Interfaces.

[63] Ying S., Yuan J., Zhang S., Sun Q., Dai Y., Qiao X., Yang D., Chen J., Ma D. (2019). High efficiency warm white organic light-emitting diodes with precise confinement of charge carriers and excitons in the exciplex host system. J. Mater. Chem. C.

[64] Zhang H., Wang Z., Gao L., Zhao B., Li W. (2018). Low efficiency roll-off and high color stability pure fluorescent white organic light-emitting diode based exciplex host. RSC Adv..

[65] Kim K.-H., Moon C.-K., Sun J.W., Sim B., Kim J.-J. (2015). Triplet Harvesting by a Conventional Fluorescent Emitter Using Reverse Intersystem Crossing of Host Triplet Exciplex. Adv. Opt. Mater..

[66] Mahmoudi M., Keruckas J., Leitonas K., Kutsiy S., Volyniuk D., Gražulevičius J.V. (2021). Exciplex-forming systems with extremely high RISC rates exceeding 10^7^ s^−1^ for oxygen probing and white hybrid OLEDs. J. Mater. Res. Technol..

[67] Zhao B., Zhang T., Chu B., Li W., Su Z., Wu H., Yan X., Jin F., Gao Y., Liu C. (2015). Highly efficient red OLEDs using DCJTB as the dopant and delayed fluorescent exciplex as the host. Sci. Rep..

[68] Lin T., Song Q., Liu Z., Chu B., Li W., Luo Y., Lee C.S., Su Z., Li Y. (2017). Effects of acceptor on the performance of exciplex-based OLED. Synth. Met..

[69] Kim H.-G., Kim K.-H., Moon C.-K., Kim J.-J. (2017). Harnessing Triplet Excited States by Fluorescent Dopant Utilizing Codoped Phosphorescent Dopant in Exciplex Host for Efficient Fluorescent Organic Light Emitting Diodes. Adv. Opt. Mater..

[70] Duan Y., Sun F., Yang D., Yang Y., Chen P., Duan Y. (2014). White-light electroluminescent organic devices based on efficient energy harvesting of singlet and triplet excited states using blue-light exciplex. Appl. Phys. Express.

[71] Zhang M., Wang K., Zheng C.-J., Liu W., Lin H., Tao S.-L., Zhang X.-H. (2017). Efficient, color-stable and high color-rendering-index white organic light-emitting diodes employing full thermally activated delayed fluorescence system. Org. Electron..

[72] Castellani M., Berner D. (2007). Competition between excitons and exciplexes: Experiments on multilayered organic light emitting diodes. J. Appl. Phys..

[73] Shakeel U., Singh J. (2018). Study of processes of reverse intersystem crossing (RISC) and thermally activated delayed fluorescence (TADF) in organic light emitting diodes (OLEDs). Org. Electron..

[74] Chen D., Xie G., Cai X., Liu M., Cao Y., Su S.J. (2016). Fluorescent Organic Planar pn Heterojunction Light-Emitting Diodes with Simplified Structure, Extremely Low Driving Voltage, and High Efficiency. Adv. Mater..

[75] Guo J., Zheng C.-J., Ke K., Zhang M., Yang H.-Y., Zhao J.-W., He Z.-Y., Lin H., Tao S.-L., Zhang X.-H. (2021). Novel triazine derivatives with deep LUMO energy levels as the electron-accepting components of exciplexes. J. Mater. Chem. C.

[76] Hung W.Y., Fang G.C., Chang Y.C., Kuo T.Y., Chou P.T., Lin S.W., Wong K.T. (2013). Highly efficient bilayer interface exciplex for yellow organic light-emitting diode. ACS Appl. Mater. Interfaces.

[77] Yersin H., Mataranga-Popa L., Li S.-W., Czerwieniec R. (2018). Design strategies for materials showing thermally activated delayed fluorescence and beyond: Towards the fourth-generation OLED mechanism. J. Soc. Inf. Disp..

[78] Wu M., Wang Z., Liu Y., Qi Y., Yu J. (2019). Non-doped phosphorescent organic light-emitting devices with an exciplex forming planar structure for efficiency enhancement. Dyes. Pigm..

[79] Liu X., Yao B., Zhang Z., Zhao X., Zhang B., Wong W.-Y., Cheng Y., Xie Z. (2016). Power-efficient solution-processed red organic light-emitting diodes based on an exciplex host and a novel phosphorescent iridium complex. J. Mater. Chem. C.

[80] Liu X.-K., Chen Z., Zheng C.-J., Chen M., Liu W., Zhang X.-H., Lee C.-S. (2015). Nearly 100% Triplet Harvesting in Conventional Fluorescent Dopant-Based Organic Light-Emitting Devices Through Energy Transfer from Exciplex. Adv. Mater..

[81] Xu T., Zhou J.G., Huang C.C., Zhang L., Fung M.K., Murtaza I., Meng H., Liao L.S. (2017). Highly Simplified Tandem Organic Light-Emitting Devices Incorporating a Green Phosphorescence Ultrathin Emitter within a Novel Interface Exciplex for High Efficiency. ACS Appl. Mater. Interfaces.

[82] Miao Y., Wang K., Zhao B., Gao L., Xu J., Wang H., Xu B. (2017). Ultra-simple white organic light-emitting diodes employing only two complementary colors with color-rendering index beyond. RSC Adv..

[83] Zhao C., Zhang T., Chen J., Yan D., Ma D. (2018). High-performance hybrid white organic light-emitting diodes with simple emitting structures and low efficiency roll-off based on blue thermally activated delayed fluorescence emitters with bipolar transport characteristics. J. Mater. Chem. C.

[84] Chao C.-I., Chen S.-A. (1998). White light emission from exciplex in a bilayer device with two blue light-emitting polymers. Appl. Phys. Lett..

[85] Zhao B., Zhang H., Miao Y., Wang Z., Gao L., Wang H., Hao Y., Li W. (2018). High color stability and CRI (>80) fluorescent white organic light-emitting diode based pure emission of exciplexes by employing merely complementary colors. J. Mater. Chem. C.

[86] Sych G., Volyniuk D., Bezvikonnyi O., Lytvyn R., Grazulevicius J.V. (2019). Dual Interface Exciplex Emission of Quinoline and Carbazole Derivatives for Simplified Nondoped White OLEDs. J. Phys. Chem. C.

[87] Wei X., Gao L., Miao Y., Zhao Y., Yin M., Wang H., Xu B. (2020). A new strategy for structuring white organic light-emitting diodes by combining complementary emissions in the same interface. J. Mater. Chem. C.

[88] Wang Z., Wang H., Zhu J., Wu P., Shen B., Dou D., Wei B. (2017). Manipulation of Thermally Activated Delayed Fluorescence of Blue Exciplex Emission: Fully Utilizing Exciton Energy for Highly Efficient Organic Light Emitting Diodes with Low Roll-Off. ACS Appl. Mater. Interfaces.

[89] Ying S., Zhang S., Yao J., Dai Y., Sun Q., Yang D., Qiao X., Chen J., Ma D. (2020). High-performance white organic light-emitting diodes with doping-free device architecture based on the exciton adjusting interfacial exciplex. J. Mater. Chem. C.

[90] Kim D.H., Cho N.S., Oh H.Y., Yang J.H., Jeon W.S., Park J.S., Suh M.C., Kwon J.H. (2011). Highly efficient red phosphorescent dopants in organic light-emitting devices. Adv. Mater..

[91] Luo D., Xiao Y., Hao M., Zhao Y., Yang Y., Gao Y., Liu B. (2017). Doping-free white organic light-emitting diodes without blue molecular emitter: An unexplored approach to achieve high performance via exciplex emission. Appl. Phys. Lett..

[92] Luo D., Li X.-L., Zhao Y., Gao Y., Liu B. (2017). High-Performance Blue Molecular Emitter-Free and Doping-Free Hybrid White Organic Light-Emitting Diodes: An Alternative Concept to Manipulate Charges and Excitons Based on Exciplex and Electroplex Emission. ACS Photonics.

[93] Xu T., Zhou J.-G., Fung M.-K., Meng H. (2018). Simplified efficient warm white tandem organic light-emitting devices by ultrathin emitters using energy transfer from exciplexes. Org. Electron..

[94] Zhang H., Liu X., Gong Y., Yu T., Zhao Y. (2021). Synthesis and characterization of SFX-based coumarin derivatives for OLEDs. Dyes. Pigm..

[95] Ouyang X., Li X.L., Ai L., Mi D., Ge Z., Su S.J. (2015). Novel “hot exciton” blue fluorophores for high performance fluorescent/phosphorescent hybrid white organic light-emitting diodes with superhigh phosphorescent dopant concentration and improved efficiency roll-off. ACS Appl. Mater. Interfaces.

[96] Wang S., Wang X., Yao B., Zhang B., Ding J., Xie Z., Wang L. (2015). Solution-Processed Phosphorescent Organic Light-Emitting Diodes with Ultralow Driving Voltage and Very High Power Efficiency. Sci. Rep..

[97] Li J., Qiao B., Zhao S., Song D., Zhang C., Xu Z. (2020). Investigation on OLEDs efficiency roll-off with interfacial charge storage and their time-resolved emission spectra. Org. Electron..

[98] Colella M., Pander P., Pereira D.S., Monkman A.P. (2018). Interfacial TADF Exciplex as a Tool to Localize Excitons, Improve Efficiency, and Increase OLED Lifetime. ACS Appl. Mater. Interfaces.

[99] Li W., Li J.Y., Liu D., Wang F., Zhang S.F. (2015). Bipolar Host Materials for High-Efficiency Blue Phosphorescent and Delayed-Fluorescence OLEDs. J. Mater. Chem. C.

[100] Shih P.I., Chiang C.L., Dixit A.K., Chen C.K., Yuan M.C., Lee R.Y., Chen C.T., Diau E.W.G., Shu C.F. (2006). Novel Carbazole/Fluorene Hybrids: Host Materials for Blue Phosphorescent OLEDs. Org. Lett..

[101] Shih P.-I., Chien C.-H., Chuang C.-Y., Shu C.-F., Yang C.-H., Chen J.-H., Chi Y. (2007). Novel host material for highly efficient blue phosphorescent OLEDs. J. Mater. Chem..

[102] Su S.J., Sasabe H., Takeda T., Kido J. (2008). Pyridine-Containing Bipolar Host Materials for Highly Efficient Blue Phosphorescent OLEDs. Chem. Mater..

[103] Jeon S.O., Yook K.S., Joo C.W., Lee J.Y. (2009). Phenylcarbazole-Based Phosphine Oxide Host Materials For High Efficiency In Deep Blue Phosphorescent Organic Light-Emitting Diodes. Adv. Funct. Mater..

[104] Ban X., Chen F., Zhao Y., Zhu A., Tong Z., Jiang W., Sun Y. (2018). Strategy for the Realization of Highly Efficient Solution-Processed All-Fluorescence White OLEDs—Encapsulated Thermally Activated Delayed Fluorescent Yellow Emitters. ACS Appl. Mater. Interfaces.

[105] Adachi C., Baldo M.A., Thompson M.E., Forrest S.R. (2001). Nearly 100% internal phosphorescence efficiency in an organic light-emitting device. J. Appl. Phys..

[106] Kang J.-W., Lee S.-H., Park H.-D., Jeong W.-I., Yoo K.-M., Park Y.-S., Kim J.-J. (2007). Low roll-off of efficiency at high current density in phosphorescent organic light emitting diodes. Appl. Phys. Lett..

[107] Lin X., Zhu Y., Zhang B., Zhao X., Yao B., Cheng Y., Li Z., Qu Y., Xie Z. (2018). Highly Efficient TADF Polymer Electroluminescence with Reduced Efficiency Roll-off via Interfacial Exciplex Host Strategy. ACS Appl. Mater. Interfaces.

[108] Miao S., Liang K., Zhu J., Yang B., Zhao D., Kong B. (2020). Hetero-atom-doped carbon dots: Doping strategies, properties and applications. Nano Today.

[109] Guo Z., Richardson J.J., Kong B., Liang K. (2020). Nanobiohybrids: Materials approaches for bioaugmentation. Sci. Adv..

[110] Gao M., Xu G., Zhang R., Liu Z., Xia H., Shao B., Xue C., Li J., Miao S., Fu W. (2021). Electrospinning Superassembled Mesoporous AIEgen−Organosilica Frameworks Featuring Diversified Forms and Superstability for Wearable and Washable Solid-State Fluorescence Smart Sensors. Anal. Chem..

[111] Xu L., Jiang X., Liang K., Gao M., Kong B. (2021). Frontier luminous strategy of functional silica nanohybrids in sensing and bioimaging: From ACQ to AIE. Aggregate.

[112] Kong B., Zhu L., Peng C., Zhang Y., Zhang W., Tang J., Selomulya C., Zhang L., Chen H., Wang Y. (2016). Direct Superassemblies of Freestanding Metal−Carbon Frameworks Featuring Reversible Crystalline-Phase Transformation for Electrochemical Sodium Storage. J. Am. Chem. Soc..

[113] Kong B., Tang J., Zhang Y., Jiang T., Gong X., Peng C., Wei J., Yang J., Wang Y., Wang X. (2016). Incorporation of well-dispersed sub-5-nm graphitic pencil nanodots into ordered mesoporous frameworks. Nat. Chem..

[114] Li M., Dai Y., Zhang Y., Wang J., Tao Y., Zheng C., Chen R., Huang W. (2020). Highly Efficient Ultrathin Fluorescent OLEDs through Synergistic Sensitization Effects of Phosphor and Exciplex. ACS Appl. Electron. Mater..

[115] Zhang D., Song X., Cai M., Duan L. (2018). Blocking Energy-Loss Pathways for Ideal Fluorescent Organic Light-Emitting Diodes with Thermally Activated Delayed Fluorescent Sensitizers. Adv. Mater..

[116] Xu T., Xie G., Huang T., Liu H., Cao X., Tang Y., Yang C. (2021). Solution-processed multiple exciplexes via spirofluorene and S-triazine moieties for red thermally activated delayed fluorescence emissive layer OLEDs. Org. Electron..

[117] Zhao J., Zheng C., Zhou Y., Li C., Ye J., Du X., Li W., He Z., Zhang M., Lin H. (2019). Novel small-molecule electron donor for solution-processed ternary exciplex with 24% external quantum efficiency in organic light-emitting diode. Mater. Horiz..

[118] Ren Q., Zhao Y., Liu C., Zhan H., Cheng Y., Li W. (2021). The exploration of acceptor ratio in thermally activated delayed fluorescent donor for the effect on exciplex OLED. Opt. Mater..

[119] Li S.-H., Wu S.-F., Wang Y.-K., Liang J.-J., Sun Q., Huang C.-C., Wu J.-C., Liao L.-S., Fung M.-K. (2018). Management of excitons for highly efficient organic light-emitting diodes with reduced triplet exciton quenching: Synergistic effects of exciplex and quantum well structure. J. Mater. Chem. C.

[120] Nakanotani H., Furukawa T., Morimoto K., Adachi C. (2016). Long-range coupling of electron-hole pairs in spatially separated organic donor-acceptor layers. Sci. Adv..

[121] Li B., Gan L., Cai X., Li X.-L., Wang Z., Gao K., Chen D., Cao Y., Su S.-J. (2018). An Effective Strategy toward High-Efficiency Fluorescent OLEDs by Radiative Coupling of Spatially Separated Electron-Hole Pairs. Adv. Mater. Interfaces.

[122] Pu Y.J., Koyama Y., Otsuki D., Kim M., Chubachi H., Seino Y., Enomoto K., Aizawa N. (2019). Exciplex emissions derived from exceptionally long-distance donor and acceptor molecules. Chem. Sci..

[123] Sheng R., Zhang E., Zhao W., Chen C., Ma W., Shao J., Duan Y., Zhao Y., Chen P. (2022). Engineering of interface exciplex system for highly efficient white organic light-emitting diodes based on single-emission-layer architecture. Org. Electron..

[124] Dong H., Jiang H., Wang J., Guan Y., Hua J., Gao X., Bo B., Wang J. (2018). High-efficiency and color-stable warm white organic light-emitting diodes utilizing energy transfer from interface exciplex. Org. Electron..

[125] Liu Y., Wei X., Li Z., Liu J., Wang R., Hu X., Wang P., Qi T., Wang Y. (2018). Interface Exciplex Anchoring the Color Stability of Solution-Processed Thermally Activated Delayed Fluorescent White Organic Light-Emitting Diodes. Adv. Opt. Mater..

[126] Grybauskaite-Kaminskiene G., Ivaniuk K., Bagdziunas G., Turyk P., Stakhira P., Baryshnikov G., Volyniuk D., Cherpak V., Minaev B., Hotra Z. (2018). Contribution of TADF and exciplex emission for efficient “warm-white” OLEDs. J. Mater. Chem. C.

[127] Cherpak V., Stakhira P., Minaev B., Baryshnikov G., Stromylo E., Helzhynskyy I., Chapran M., Volyniuk D., Hotra Z., Dabuliene A. (2015). Mixing of phosphorescent and exciplex emission in efficient organic electroluminescent devices. ACS Appl. Mater. Interfaces.

[128] Wang H., Dong D., Lian L., Zhu F., Xu D., Wu S., He G. (2019). High Efficiency Non-Doped White Organic Light Emitting Diodes Based on a Bilayer Interface-Exciplex Structure. Phys. Status Solidi A.

[129] He Z., Wang C., Zhao J., Du X., Yang H., Zhong P., Zheng C., Lin H., Tao S., Zhang X. (2019). Blue and white solution-processed TADF-OLEDs with over 20% EQE, low driving voltages and moderate efficiency decrease based on interfacial exciplex hosts. J. Mater. Chem. C.

